# Supplementary material for: Early ethics: Exploring moral intuition and maternal Influence in preschool children
Source: PLoS One. 2026 Jan 30;21(1):e0337474. doi: 10.1371/journal.pone.0337474 (PMC12857926; doi:10.1371/journal.pone.0337474)
Supplement: Attachment — Submitted filename: Review (1).docx [file pone.0337474.s002.docx]

**TITLE: PRESCHOOLERS PARTIALLY RESPOND TO MORAL DILEMMAS AS THEIR MOTHERS DO**

**REVIEW**

***Introduction part***

The topic is very significant and up to date in the world that was so much affected by pandemics. However, there is a lack of need and significance substantiation of this research (in this paper).

The title of the article does not seem to clearly reflect the whole idea and is it more of a sentence rather than title. The description deontological principles should also have a place in the data analysis section showing the principles’ connection with the emerged dilemmas.

The small theoretical part seems very undeveloped and lacks more substantiation of moral dilemmas and their coherence with the deontological principles in data analysis. The analysis of the moral dilemmas origin and reasons why they emerge would add a value to the literature review. The problems parents experience at work should have an impact on moral dilemmas between mothers and children, as all experiences in certain environments may have various effects, especially in the case of destructive experiences. This topic is researched by the authors, that should be considered in the literature review part: Veinhardt and Deikus (2021) - <https://www.frontiersin.org/journals/psychology/articles/10.3389/fpsyg.2021.702284/full> ; Nyx L. Ng, Dillon M. Luke, and Bertram Gawronski (2025) - <https://journals.sagepub.com/doi/pdf/10.1177/01461672231180760> and others.

While the theoretical dilemma definitions were offered, there is a lack of clarity on the applicability of the results of this study. There is a lack of evidence on how moral dilemma response similarity between mother and children’s dyads can implicate chronic disorders. Psychological disorders are not caused by one factor. It is a strong encouragement to the authors to make this interlink clearer using and going back to the review of literature.

Also, consider the following questions for the review:

- How can moral dilemmas predict mental health challenges?
- What is the relationship between moral dilemma congruence between mothers and children AND mental health challenges?

***Material and Methods part***

First, there is a lack of methodological approach. There is an ambiguity in case research description: there is a need for clarification on research data collection and analysis methods in accordance with the selected methodological approach. Particularly, authors do not describe the data collection instruments, whether it is a survey or other, what sources these instruments were based on, etc. Later the authors present tests, which lack of description and who created them and what was the literature basis for that creation. The authors present statistical analysis with the sample 56, which is very small, and it is doubtful whether the results can be admitted as statistically significant. The other issue is that the authors did not substantiate the sample as reliable – literature sources should be used to prove that.

***Ethical Considerations part***

Since a sensitive topic is being analysed, it is important to describe all necessary ethical issues for vulnerable groups and topics so that research ethics should not be violated, especially as children (3-6 years) were separately researched. It should definitely be more elaborated. Children hearing about a trolley problem in those ages is exposure to violence. If children were subject to various topics such as the trolley problem, it is rather exposing to latent violence by train. How was this addressed? Were there any safeguarding measures for children participating?

***Application Procedure part***

Test procedures were completed during the data collection. The need for more detailed clarification about tests is vital:

- Did authors use 2 tests, or it was the same for mothers and children (even if children had stories read by trained persons).
- Still, it is not clear how the data was collected – whether the participants had to write answers or tell them? Also, there is not any relation to statistical analysis at this point. Were the participants asked some sort of questions, if yes, what are they? This is before control questions.
- Two control questions were asked, but the authors did not indicate who they asked to answer them – mothers or children.

***Statistical Analysis and Results parts***

The authors present permutation test, ż test, Kappa Statistic and Spearman’s correlation, McNemar’s test in this part, but it is important to explain why they are used for and give proof from other sources. The result part needs to be more detailed in explanations.

***Discussion part***

Tables and figures are not usually used in the discussion part. In the limitation section the authors say that interviews were caried out. So, this is a huge misunderstanding of methodological approaches – interviews are qualitative methods and are not used in statistical analyses. 56 sample is too small for statistics (even though it is convenience sampling); therefore, it is a strong recommendation to think of a different and very clear methodological approach. An in this part it seems that trained applicators for both mothers and children, while in the methods part it is different.

The limitations’ part should be expanded, and further research issues should be discussed for practical and theoretical implications.

**REVIEW CONCLUSION**

*In general*, the topic itself suggests a qualitative methodological approach. But if it is still going to be based on statistics (which is very doubtful in this case), then the whole methodology part needs more substantiation and proof from the previous studies and sources, as well as sample expansion.

Also, a more technical comment. The article needs further refining on the use of the English language. Please consider using language editing services to refine the manuscript. I.e., we cannot use shortened words “didn’t” for “did not” in academic writing. We typically use the full phrases “did not”, “cannot” and so on.

*Considering all the above*, major corrections should be completed.
