# Supplementary material for: Early ethics: Exploring moral intuition and maternal Influence in preschool children
Source: PLoS One. 2026 Jan 30;21(1):e0337474. doi: 10.1371/journal.pone.0337474 (PMC12857926; doi:10.1371/journal.pone.0337474)
Supplement: Attachment — Submitted filename: EARLY ETHICS.docx [file pone.0337474.s004.docx]

**EARLY ETHICS: EXPLORING MORAL INTUITION AND MATERNAL INFLUENCE IN PRESCHOOL CHILDREN**

Thank you for revising the manuscript, but it still has very confusing parts that need to be revised with more accurate attention.

**First of all**, the introductory part, even though it was expanded, but the reader is could be confused, as the authors did not highlight the main parts: topicality, research aim and the research problem. The reader is left by himself/herself to figure that out. The authors should clearly state these issues so that everyone has the same understanding.

**Second**, the methodological part is not clarified enough – it lacks theoretical substantiation and citations of scientific literature. This part is very complicated if we need to duplicate. There should be clearly stated and described (with scientific literature substantiation) these missing issues:

- Research data collection methods;
- Research data analysis methods;
- Sample selection criteria and methods;
- Etc.

I strongly recommend the authors look through other high quality published articles and see how they are constructed. Also, authors should use clear and unified terms to make the idea and text as clear as possible (e.g., “The first control case…”, but “The second control scenario“ lines – 47-53). Is “the case” and “the scenario” the same? It raises misunderstandings.

**Third**, the discussion part still seems weak. The authors should clearly state: implications for theory, implications for practice, limitations and further research. The authors should come back and check for these issues, highlighting them. Also it is essential to expand on limitations (it is important to use critical thinking) and on further research, which is way beyond qualitative research. As soon as the authors accurately highlight implications for theory and practice, the limitation section will be clear to them, and the accurately written limitations part will help to highlight further research. These issues are interconnected.

**In general**, all-important issues should be highlighted in the text so that readers can easily find them. It is recommended for the authors to stand in the readers shoes and imagine if they were the readers, reading the study for the first time, would it be easy to duplicate it. To this point, the manuscript must be revised once more and cannot be published as it is.
