# Supplementary material for: Early ethics: Exploring moral intuition and maternal Influence in preschool children
Source: PLoS One. 2026 Jan 30;21(1):e0337474. doi: 10.1371/journal.pone.0337474 (PMC12857926; doi:10.1371/journal.pone.0337474)
Supplement: Attachment — Submitted filename: Response_to_Reviewers_auresp_2.docx [file pone.0337474.s005.docx]

**Response to Reviewers**

Dr. Emily Chenette

Editor in Chief

**PLOSE ONE Journal**

On behalf of my co-authors and myself, I am resubmitting the manuscript entitled *“Preschoolers Partially Respond to Moral Dilemmas as Their Mothers Do”* to be considered for publication in the PLOS ONE Journal.

We have carefully revised the manuscript, taking into account all the reviewers' suggestions. You will find in the following text detailed responses to the reviewers’ recommendations and comments, along with the relevant changes made to the manuscript, which are directly reported in our answers. Finally, a marked-up version of the manuscript with all changes is provided.

We hope the revised manuscript meets the reviewers’ expectations and can be accepted for publication; otherwise, we are open to further improvements.

We appreciate the opportunity to consider our work.

Sincerely,

María G. Jean-Tron, Juan Garduño-Espinosa*, Diana Ávila-Montiel, Gina C. Chapa-

Koloffon, Oscar A. Resendez-Berber, Elizabeth Cruz-Cruz, Guillermo Salinas-

Escudero, and Onofre Muñoz-Hernández.

*Correspondence

Juan Garduño-Espinosa: [juan.gardunoe@gmail.com](mailto:juan.gardunoe@gmail.com)

**Reviewer 1**

*Thank you for revising the manuscript, but it still has very confusing parts that need to be revised with more accurate attention. First of all, the introductory part, even though it was expanded, but the reader is could be confused, as the authors did not highlight the main parts: topicality, research aim and the research problem. The reader is left by himself/herself to figure that out. The authors should clearly state these issues so that everyone has the same understanding.*

Answer: The requested changes have been made to make the Introductory part more straightforward.

*Second, the methodological part is not clarified enough – it lacks theoretical substantiation and citations of scientific literature. This part is very complicated if we need to duplicate. There should be clearly stated and described (with scientific literature substantiation) these missing issues: • Research data collection methods; • Research data analysis methods; • Sample selection criteria and methods; • Etc. I strongly recommend the authors look through other high quality published articles and see how they are constructed.*

Answer: Thanks for the comment. The Methodology section was restructured, aiming to make it more understandable and detailed. The "Sample selection criteria and methods" were detailed in the "participants" section on page 6; the sample size calculation was also included, along with references (page 6). The "Research data collection methods" is detailed in the "Application procedure" section (page 9), and the "Research data analysis" is detailed in the "statistical analysis" section (page 11), and references were added in this part as well.

*Also, authors should use clear and unified terms to make the idea and text as clear as possible (e.g., “The first control case…”, but “The second control scenario“ lines – 47- 53). Is “the case” and “the scenario” the same? It raises misunderstandings.*

Answer: Indeed, "the case" and "the scenario" refer to the same in the manuscript; to avoid confusion, we homogenize the terms throughout the manuscript.

*Third, the discussion part still seems weak. The authors should clearly state: implications for theory, implications for practice, limitations and further research. The authors should come back and check for these issues, highlighting them. Also it is essential to expand on limitations (it is important to use critical thinking) and on further research, which is way beyond qualitative research. As soon as the authors accurately highlight implications for theory and practice, the limitation section will be clear to them, and the accurately written limitations part will help to highlight further research. These issues are interconnected.*

Answer: According to the commentary, it began with a general discussion, then addressed the theoretical and practical implications (page 19), and then outlined the limitations and future research (page 22). We consider that with the new information, the discussion will be more nourished and clear and will give rise to new research.

*In general, all-important issues should be highlighted in the text so that readers can easily find them. It is recommended for the authors to stand in the readers shoes and imagine if they were the readers, reading the study for the first time, would it be easy to duplicate it. To this point, the manuscript must be revised once more and cannot be published as it is.*

Answer: Thank you for the comment, it's very accurate. We have highlighted all the essential parts, hoping that the text in its entirety will be more complete and understandable.

**Reviewer 2**

*Many of the responses given are acceptable. However, a number of further points have arisen. The most serious, it seems to me, is the potential contamination of the data by the children being able to see (and hear) their mothers. I am not satisfied that this has been appropriately reported in the manuscript. Please see my further comments on it below and other issues that have arisen:*

*3: “Moral dilemmas arise when two or more of these principles come into irresolvable conflict.” Are moral dilemmas irresolvable? Why do you need this claim? I suggest removing the word ‘irresolvable’.*

Answer: Okay, thanks for the observation. The change has been made (p. 3).

*4: It is uncertain what if anything has changed in the red paragraph from the first version. The authors mention a paper by ‘Green[e] et al.’, but there is no reference for it. “through moral reasoning about costs and benefits, a utilitarian judgment can be reached, while judgments based on norms (deontological) would be through automatic emotional reactions (moral intuitions) (19).” “automatic emotional reactions (moral intuitions)” This is not generally what ‘moral intuition’ means the ethical literature. Further, the authors are misrepresenting what is said by Greene (19). What they actually say is: “Finally, there is the view for which I will argue, that deontology is more emotionally driven while consequentialism is more “cognitive.” I hasten to add, however, that I don’t believe that either approach is strictly emotional or “cognitive” (or even that there is a sharp distinction between “cognition” and emotion). More specifically, I am sympathetic to Hume’s claim that all moral judgment (including consequentialist judgment) must have some emotional component (Hume, 1978)” [p. 41] So, please revise your statement carefully to reflect accurately the nuance of Greene's position.*

Answer: Thank you for the comment. The literature was reviewed again, and the paragraph was restructured to avoid confusion about what Green said in his theory. The quote is included (page 4). We agree with your view that cognitive and emotional processes cannot be separated and that both can be found in both utilitarian and deontological judgments, so we also include some of Greene and Haidt's theory within the discussion, in the section on theoretical implications (p. 19).

*9: “Mothers and children were in separate areas within the waiting room, so they could neither hear nor see each other.” In your response to my question the authors say: “We tried to get the child to focus on the interviewers so that he wouldn't hear or see his mother. We consider that the answers could be altered if the child saw his mother.”These are two distinct descriptions of the experimental situation. In the manuscript the authors say that it is not _possible_: ‘could neither hear nor see each other’. However, it clearly was possible because the child would see (or hear) their mother if the interviewer did not manage to keep their focus, as you say in your response to my question.It is important that you provide the reader with an accurate picture of the exact experimental conditions. Was any measure taken of how much each child focused or did not focus? If not, then how do the authors know that the answers were not influenced, or the data not contaminated? This is potentially quite problematic for the study. Please consider this carefully, and provide your reader with the full and accurate information and an assessment of the effect that it did or could have had on the results.*

Answer: An apology for the confusion; our previous answer was not properly structured. In effect, children were separated from their mothers when asked to address ethical dilemmas. The comment about keeping their attention on the interviewer referred to them not worrying about not seeing or hearing their mother and wanting to look for her. Of course, before separating them, they were told what would be done and where their mother would be while they met with the interviewers. It is briefly explained in the "Application Procedure" section on page 10.
