# Supplementary material for: Early ethics: Exploring moral intuition and maternal Influence in preschool children
Source: PLoS One. 2026 Jan 30;21(1):e0337474. doi: 10.1371/journal.pone.0337474 (PMC12857926; doi:10.1371/journal.pone.0337474)
Supplement: Attachment — Submitted filename: REVIEWER COMMENTS.docx [file pone.0337474.s006.docx]

REVIEWER COMMENTS

Thank you for addressing the comments, but the methodological part is still not clear enough. The "dyad" concept was not properly explained. In previous research a dyad is likely 2 participants, but the the authors research seems to be one. This is not good to have the misunderstanding, should be definitely clarified. Also to substantiate the research problem only one source is used. It can not be like that. Methods part needs more attention according to methodological principals that are general rules for all. Thank you
